# A Comparison of Motives by Gender and Age Categories for Training at Norwegian Fitness Centres

**DOI:** 10.3390/sports9080113

**Published:** 2021-08-21

**Authors:** Stian Larsen, Tarron Mozdoorzoy, Eirik Kristiansen, Hallvard Nygaard Falch, Tore Kristian Aune, Roland van den Tillaar

**Affiliations:** Department of Sport Sciences and Physical Education, Nord University, 7600 Levanger, Norway; stianandrelarsen@live.no (S.L.); tarron92@hotmail.com (T.M.); ek1105@hotmail.com (E.K.); falch7@hotmail.com (H.N.F.); tore.k.aune@nord.no (T.K.A.)

**Keywords:** strength training, apparel, enjoyment, vitality, intrinsic and extrinsic motivations

## Abstract

Examining participatory motives clarifies what engages and keeps individuals participating in exercise. The popularity of training at fitness centres has greatly increased over the last two decades, but individual determinants for motivation remain uncertain. This study compared motives between gender and age categories in training and performing physical activity at Norwegian fitness centres. To compare motives, a survey utilising a standardised questionnaire (MPAM-R) was conducted at six different Norwegian fitness centres. It was hypothesised that the intrinsic motive socialisation and extrinsic motive fitness would be more important among the older age categories for both genders, while the extrinsic motive appearance and intrinsic motive enjoyment would be more important among younger age groups. A total response of 183 men and 150 women, aged 14–80 years, was divided into seven categories based on their age and included in the statistical analysis. The main findings after conducting a two-way analysis of variance (ANOVA) with repeated measures, were that the most important motive for training at fitness centres was increasing fitness, followed by enjoyment, competence, vitality and appearance. The social motive was rated the lowest. Women rated fitness and enjoyment higher compared to men, and men rated the motive for appearance higher than women, but this decreased with age in both genders. With increasing age, the importance of enjoyment and competence decreased in men, while women seemed to place increased importance on vitality with age. The importance of the social motive decreased first as age increased, but then increased again in the age group 41–50 years and older. It was concluded that the motives for participating in exercise at fitness centres was dependent on individual characteristics and that motives about training at fitness centres differed by gender and changed with age.

## 1. Introduction

Participation in different types of physical activity is associated with health benefits and reduction in chronic diseases [1], but these benefits are not maintained without adherence through regular participation [2,3]. Maintaining adherence may be attributed to the underlying motives for performing physical activity. Several studies have reported miscellaneous motives for physical activity including fitness, enjoyment, competence, socialising, appearance, and vitality, which have all been reported as important motives for performing diverse types of physical activity in the forms of sports and exercise participation in different cohorts [4,5,6,7].

Researchers have concluded that these motives could be a function of extrinsic or intrinsic factors [8,9], while the self-determination theory (SDT) developed by Deci and Ryan [10] provides a framework for understanding exercise motivation for participating in physical activity. The cognitive evaluation theory, a subtheory of SDT, is often used to explain exercise motivation [4]. This subtheory suggests that exercise motivation could be either extrinsic or intrinsic: extrinsic variables may explain motivation for outcomes extraneous to the participation in the exercise itself, while intrinsic motivation is more closely related to competence and the exercise itself [4,11]. According to this theory, extrinsic motivation can be related to different motives such as fitness, socialising and appearance, whereas intrinsic motivation can be related to motives such as enjoyment, competence and vitality.

Egli, Bland, Melton and Czech [4] examined differences in exercise motivation between genders for college students and found that men tended to be motivated by intrinsic factors such as competition and challenge, whereas women tended to be motivated by extrinsic factors such as weight management and appearance. Kilpatrick et al. [12] have compared motivation for sports versus exercise participation among college students, and they reported that intrinsic motives, such as enjoyment, were more likely to be reported in sports participation, whereas extrinsic motives such as appearance and fitness were more likely to be reported in the exercise group. The authors therefore suggested that sports participation could be more beneficial to ensure adherence in physical activity than exercise participation in a fitness centre. Moreover, Quesada et al. [13] compared different motivational profiles who did non-competitive physical sports activities at a public sport centre in Spain. The authors found three motivational profiles, whereas the social aspect of training was most important among the adults who preferred performing physical activity at the pool. The second profile was the most motivated, which trained for competitions factors such as health and fun. The last motivation profile was motivated by physical appearance and less health and competence motives. Those were young males, whose favourite area for performing physical activity was in the muscle building room.

In the past few decades, resistance exercises, cardiovascular exercises (among others) and participation in group workouts in fitness centres have increased in popularity [14]. In Norway, the first fitness centre opened in the 1950s in Oslo [14] and was already commercial during the first decade [15]. According to Ommundsen and Aadland [16], approximately 8% of the Norwegian population exercised at a fitness centre in 1987, while approximately 30% of the Norwegian population performed a workout at a fitness centre in 2018 according to Virke [17]. A yearly statistical survey in Norway about living terms for the Norwegian population showed that around 80% of the Norwegian population exercised at least once a week in 2019 [18].

The report by Støren and Lundgaard [18] also revealed that both genders and age categories affect what type of workout the person performs. While women often participated in both group lessons and resistance exercises, men more often reported that they performed jogging as an exercise during the last 12 months [18]. Among the age categories 16–24, 25–44, 45–66 and >67 years, 10.7%, 2.9%, 1.6% and 2%, respectively, reported that they have performed some type of resistance exercise in the last 12 months. Body mass regulation together with appearance-related motives for performing physical activity have seen more than a 50% increase from 1989 to 2015, according to Breivik and Rafoss [19]. A national survey conducted by Barland and Tangen [20] on physical activity and appearance in adolescents revealed that approximately 91% of Norwegian 18–19 year olds used fitness centres as an arena for achieving a better appearance. Breivik and Rafoss [19] also reported that enjoyment was a more frequently reported motive for the younger age categories when participating in exercise and physical activity, and women reported more frequently that they exercised to improve health 

Despite these findings, to the authors’ knowledge, no studies have investigated the underlying motives for performing physical activities at fitness centres among different age categories and between genders in Norway. Investigating this could explain why both gender and age categories reported divergent participation in different exercises [18]. These differences may be explained by the underlying motives for performing physical activities. This study therefore compared motives between genders and age categories for training and performing physical activity at Norwegian fitness centres. It was hypothesised that the intrinsic motive socialising and the extrinsic motive fitness would be more important among the older age categories for both genders, while the extrinsic motive appearance and the intrinsic motive enjoyment would be more important among younger age groups. Additionally, it was hypothesised that appearance would be more important among women than men.

## 2. Materials and Methods

### 2.1. Method

To compare differences in motives by gender and age categories for training at Norwegian fitness centres, a questionnaire survey addressing motives for exercising was carried out at different Norwegian fitness centres in the spring of 2018. The fitness centres in which responses were obtained, are commercial and unisex with prices ranging from low-cost to high cost. The standardised questionnaire Motives for Physical Activity Measure–revised (MPAM-R), containing 30 items, was used; this questionnaire evaluates the five following motives for physical activity: enjoyment, fitness, competence, social and appearance.

According to Richard, Christina, Deborah, Rubio and Kennon [7], fitness refers to being physically healthy/active, energetic and strong. Appearance refers to becoming physically more attractive, looking better and reaching or maintaining a desired body mass. Competence refers to being physically active because of the need to improve at an activity, acquiring new skills and facing a challenge. Social refers to being with friends and meeting new people while exercising. Enjoyment refers to being physically active simply because it is interesting, fun, enjoyable and makes one happy. The MPAM-R is a longer, revised version of an earlier scale by Frederick and Ryan [5] and has been validated and used in research on intrinsic motivation, motive in sports and physical activity [7,21,22,23,24,25]. The subscale-scores for the MPAM-R show high internal consistency (Cronbach’s Alpha = 0.870). Answers to items in the questionnaire are on a seven-point Likert-type scale, where 1 is defined as not a reason, while 7 represents the most important reason. 

### 2.2. Participants

Data were collected from 350 participants from different fitness centres in Norway, aged 14–80 years, which were divided into seven predetermined age-categories based on their age. Detailed information regarding the purpose of the study was included along with the questionnaire. The Regional Committee for Medical and Health Research Ethics waives the requirement for ethical approval for such studies. Therefore, the ethics of the study was completed according to the institutional requirements and approval for data security and handling was obtained from the Norwegian Centre for Research Data. The study was conducted in line with the current ethical regulations for research and with the latest Declaration of Helsinki. 

### 2.3. Procedures

To gain permission to carry out the study at the different fitness centres, the CEOs of the fitness centres were contacted to explain the objective of the current study. Seven fitness centres were contacted, and the questionnaire was distributed to members of six different fitness centres. After gaining permission, the questionnaires were strategically placed at the entrance of the different fitness centres. The respondents could thus participate anonymously, which hopefully promoted honest responses and increased the validity of the study. Questionnaires that were not fully answered were excluded from the analysis. Furthermore, participants needed to have at least 6 months training experience in a training centre prior to the questionnaire to be included in the analysis. In total, 350 questionnaires were collected, of which 335 were fully answered. Two more questionnaires were removed due to a small sample size within that age category (80<), thus reducing the total number of surveys to 333 responses included in the statistical analysis. Of the 333 questionnaires which met the inclusion criteria, responses were from 183 men and 150 women (Table 1).

### 2.4. Statistical Analysis

To identify differences in motives among gender and age categories a 6 (motives: repeated measures) × 2 (gender) × 7 (age groups) analysis of variance (ANOVA) was carried out. If interactions were found, two-way ANOVAs by motive, gender and among age categories were also conducted to identify differences between men and women and the seven age categories. If the analyses revealed significant differences, post hoc tests with Holm Bonferroni correction were performed. Results in the tables are presented as mean ± standard deviations. Effect sizes were evaluated with η^2^ (partial eta squared), where <0.01–0.06 constitutes a small effect, 0.06–0.14 a medium effect, and >0.14 a large effect (Cohen, 1988). The alpha level of significance was set at *p* < 0.05. Statistics were analysed in SPSS Version 27.0 (IBM Corp., Armonk, NY, USA). 

## 3. Results

**Figure 1:**
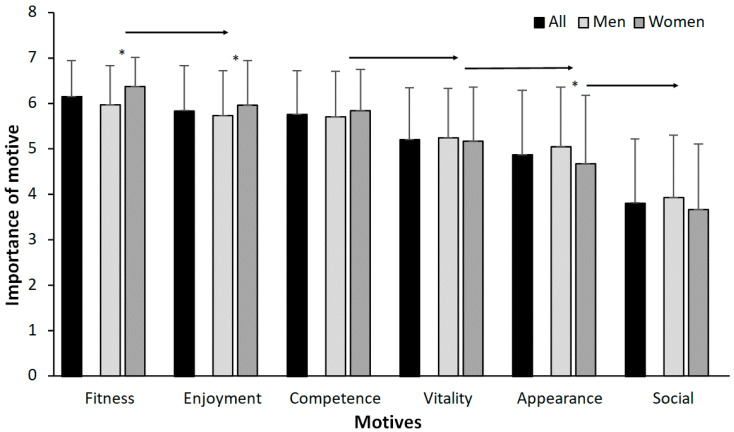
Mean ± SD of importance for the different motives averaged overall and per gender. * Indicates a significant difference between genders for this motive on a *p* ≤ 0.05 level. → indicates a significant difference between this motive and all right of the arrow for both genders on a *p* ≤ 0.05 level.

**Figure 2:**
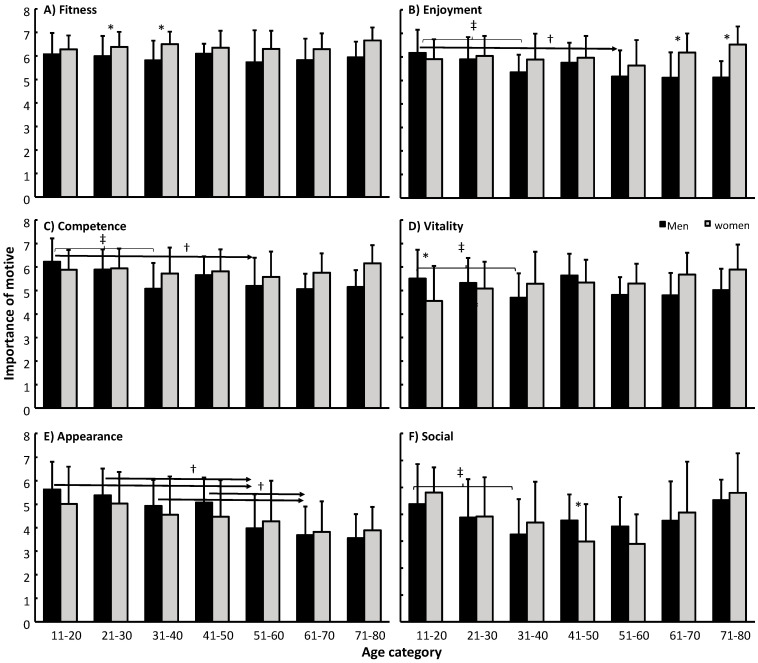
Mean ± SD of importance of motives for each motive per age group and gender. * Indicates a significant difference between genders for this motive on a *p* ≤ 0.05 level. ‡ indicates a significant difference between these two age groups for this gender on a *p* ≤ 0.05 level. † indicates a significant difference between this age group and all right of the arrow for this gender on a *p* ≤ 0.05 level.

There was a clear priority in the motives when the data for all subjects (F = 298, *p* < 0.001, η^2^ = 0.47) were analysed. The most important motive for training at a fitness centre was increasing fitness. Enjoyment and competence were rated equally, but lower than fitness, followed by vitality and appearance. The social motive was rated the lowest (Figure 1). When comparing the motives specified between men and women, women rated fitness and enjoyment higher than men did, and men rated the appearance motive higher than women did (Figure 1). Post hoc comparison by age group showed that women rated fitness as more important than men did between in the age range 21–40 years and enjoyment from 61 years and older (Figure 2A,B). Only men between 11 and 20 years old rated vitality and men aged 41–50 years rated the social motive higher than women in the same age group (Figure 2D,F).

The importance of the different motives changed with age (F = 4.1, *p* < 0.001, η^2^ = 0.071) and by gender over age (F = 2.7, *p* = 0.013, η^2^ = 0.05). When analysed by motive, it appeared that enjoyment, competence, social and appearance changed significantly with age (F ≥ 2.2, *p* ≤ 0.040, η^2^ ≥ 0.04) and that there was significant interaction effect of gender × age for the motives: enjoyment, competence and vitality (F ≥ 2.1, *p* ≤ 0.047, η^2^ ≥ 0.04). Post hoc comparison revealed the importance of enjoyment and competence decreased in men with age, while no changes occurred in women, thereby creating a gender × age interaction effect (Figure 2B,C). The importance of appearance also decreased with age in both genders (Figure 2E), but only men showed significant decreases (F = 8.0, *p* < 0.001, η^2^ = 0.13). Vitality decreased for men between the age groups 11–20 with 31–40 years, while women seemed to feel an increased importance for vitality with age, thus causing the interaction effect (Figure 2D). The importance of the social motive decreased first with increasing age, but then increased again from the group 41–50 years and older (Figure 2F).

## 4. Discussion

As a first step in understanding what keeps and engages individuals participating in exercise involves examining their participatory motives. Distinct characteristics between individuals might lead to dissimilar motives for exercise [26], and the simplest distinctions probably rest on gender and age. The present study compared the effect of gender and age upon the motives for exercise in a fitness centre, and the present results revealed several gender and age effects. The main findings were that the most important motive for training at a fitness centre was increasing fitness, followed by enjoyment and competence, vitality and appearance. The social motive was rated the lowest by all respondents. Women rated fitness and enjoyment higher than men did, and men rated the motive for appearance higher than women did, but this decreased with age in both genders. With increasing age, the importance of enjoyment and competence decreased in men, while women placed increased importance on vitality with age. The importance of the social motive at first decreased with increasing age, but then increased again in the age groups 41–50 years and older.

The present study showed a substantial priority between different motives. In total, for all participants, the most important motive for exercising at a fitness centre was increasing fitness, and the present data suggest the main motive for participating in a training centre independent of age and gender is due to a desire to increase physical fitness. Further analysis showed that women rated fitness higher than men did, and comparisons by age group showed that women rated fitness as more important than men in the age categories between ages 21–40 years, which was in line with the findings of Molanorouzi, et al. [27] on physical activity. The underlying explanations for why fitness is highly ranked as a motive might be the health benefits, health pressure from society, ill-health avoidance and more body-related motives such as weight management.

The second most significant motives, in total, that respondents reported for exercising at a fitness centre were enjoyment and competence, and these two motives were rated equally. Most participants identified exercise as enjoyable, and the intrinsic motivation likely promotes regular attendance and participation in physical activity and exercise at a fitness centre. The overall results for gender differences showed that women also rated enjoyment and competence higher than men did. Additionally, comparisons by age group showed that women rated enjoyment as more important than men in the age groups from 61 years and older. That men rated competence lower than women, which was in contrast to earlier studies [28,29]. This could indicate that women are becoming more interested in increasing their competence by training, as higher competence may be why they are training certain exercises or structure their training plan. Increased competence may correlate with enjoyment: as women gain more knowledge within training, they may feel more enjoyment in training at a fitness centre than men do.

In total, vitality and appearance were reported as more important for men than women in exercising at fitness centres. Further analysis showed that men rated vitality and appearance higher than women did and post hoc comparisons per age group showed that only men aged 11–20 years rated vitality and appearance higher than women of the same age. The vitality motive decreased for men from the 11–20 age group to the 31–40 age group, while women seemed to place an increased importance on vitality with age. The importance of appearance decreased with age and gender, but only men showed significant decreases. This contrasts with previous research on gender, which found that men had lower scores than women for extrinsic motives related to appearance [4]. This may indicate that there is a shift been genders, where the motivation for training in early age for men is more extrinsically motivated, which may arise from the social demands to achieve lean body types and a youthful appearance being greater for men than for women. This speculation is supported by Quesada et al. [13] who found that young men were less motivated by health and competence motives, but more motivated by physical appearance, and that they favoured performing exercises in the muscle-building room. 

The social motive was, in total, rated as the least important motive for exercising at a fitness centre. Vlachopoulos, Asci, Cid, Ersoz, González-Cutre, Moreno-Murcia and Moutão [26] stated that women are more often motivated to engage in exercising due to social interaction compared to men, but this assumption was not confirmed in the present study. The importance of the social motive decreased after age 11–20 but increased again for those aged 41–50 years and older. The only significant difference between genders was that men between the ages of 41–50 years rated the social motive higher than women of the same age, which is again in contrast to the study by Vlachopoulos, Asci, Cid, Ersoz, González-Cutre, Moreno-Murcia and Moutão [26]. These results demonstrate that young men and women are socialising in fitness centres, then their motives change for a period, before social motives for exercising increases again from the age of 60 years and older.

What drives people to exercise and engage in physical activity is one of the most frequently asked questions by exercise professionals and peers. Understanding the motives for individual exercise could be of great practical value [30], and research on the design of fitness centres and the conceptualisation of exercise programmes according to motivation is essential. In an empirical manner, the results showed that motives could differ by gender and that the motives for exercise change by age group [30]. The first time a person engages in physical exercise may be based primarily on one motive, but as the person ages, other motives may arise. Future studies should analyse the reasons for the practice of exercise over different age spans and by gender. People may have very individualised motives for exercise, and professionals should be aware of and support those individual reasons and not impose general conceptualised programmes for training. Fitness centres and exercise professionals should be attentive to individual needs and be curious about their motives. In general, according to SDT [10], it could be an advantage when motivating people who are exercising for extrinsic motives (e.g., for social recognition) to help them discover intrinsic motives. It is, however, necessary to keep in mind that people can exercise based on both extrinsic and intrinsic motives, and that this might be constructive in some instances. Moreover, since the most important reason for exercising at fitness centres is increasing fitness, it is speculated that a significant percentage of people choose to exercise because of controlled motivations, based on a feeling of having to exercise rather than wanting to exercise [31]. Such controlled form of motivation is not autonomous [32]. An arisen hypothesis is that since the external motive fitness was the most important, this could be partially responsible for the dropout rate at Norwegian fitness centres.

The present results showed some similarities between genders in terms of motives for exercising at fitness centres; however, the present results also show several differences between men and women. Therefore, understanding why men and women engage in exercise could be of the upmost importance. The overall results also showed that the importance of motives changes throughout different age spans and between genders over time. In general, the importance of enjoyment, competence and appearance decreases with age, especially in men, while the social motive follows a U-shape, with a high impact in early age, a decrease in middle age and then an increase from 60 years and above. Since the intrinsic motives enjoyment and competence decreased with age, which is important for training adherence [31], it is speculated that this could be an explanation for why there were less participants in the older age categories. 

The present study has some limitations. The total number of respondents was 350, but when divided into gender and age categories, the number of respondents in each group was quite small and partly unbalanced for the respective groups, which could influence the results. Further studies with a larger sample would make it possible to draw bolder conclusions. Despite this limitation, several interesting and significant differences in both gender and age spans were documented in the present study, which gives a good indication about the motives for training in a fitness centre. This study was conducted in Norway at only six fitness centres, which may have its own idiosyncratic culture for training at fitness centres compared to other countries, so the results may not be generalisable. Moreover, it has been reported among women that the motives for performing physical activities at fitness centres may differ between mixed centres and those who go to exclusive female centres [33]. Therefore, future studies should be conducted in other countries and at exclusive female and male fitness centres to determine if the motives for training at fitness centres are similar in these occasions.

## 5. Conclusions

The motives for participating in exercise at fitness centres depend on individual characteristics that have both gender and age effects. However, our findings suggest that the motive appearance is of greater importance for men than women, whereas fitness and enjoyment are more important for men, but the importance of these motives becomes less important with age for both genders. Moreover, the motives enjoyment and competence are of minor importance for the older age categories among men. Additionally, an important motive for exercising at fitness centres among older women are vitality, whereas the social motive is of greater importance among the older age categories independent of gender. It could be beneficial for staff at fitness centres to have these differences in mind when designing training programmes and facilities for a population of mixed gender and age groups. More studies are needed to assess the differences in other characteristics (e.g., economic status or low-cost vs. premium fitness centre), and future studies will increase our knowledge about the individualised motives for pursuing physical fitness activities at training centres.

## Figures and Tables

**Table 1:** Frequency table of number (*n*) of men and women within each age category.

Age (Years)	11–20	21–30	31–40	41–50	51–60	61–70	71–80
Women	21	53	22	23	15	11	5
Men	40	69	22	19	11	10	12

## Data Availability

The data presented in this study are available on request from the corresponding author. The data are not publicly available due to rules of Norwegian Center for Research Data.
